# Remote pulmonary rehabilitation for interstitial lung disease: developing the model using experience-based codesign

**DOI:** 10.1136/bmjresp-2023-002061

**Published:** 2024-02-20

**Authors:** Lisa Jane Brighton, Nannette Spain, Jose Gonzalez-Nieto, Karen A Ingram, Jennifer Harvey, William D-C Man, Claire M Nolan

**Affiliations:** 1 Cicely Saunders Institute of Palliative Care, Policy and Rehabilitation, King's College London, London, UK; 2 Department of Psychology, King's College London, London, UK; 3 CREATE-ILD Patient and Public Involvement Group, Guy's and St Thomas' NHS Foundation Trust, London, UK; 4 Harefield Pulmonary Rehabilitation Unit, Guy's and St Thomas' Hospitals NHS Trust, London, UK; 5 Harefield Respiratory Research Group, Guy's and St Thomas' Hospitals NHS Trust, London, UK; 6 National Heart and Lung Institute, Imperial College London, London, UK; 7 Faculty of Life Sciences and Medicine, King’s College London, London, UK; 8 College of Health, Medicine and Life Sciences, Department of Health Sciences, Brunel University London, London, UK

**Keywords:** Interstitial Fibrosis, Pulmonary Rehabilitation

## Abstract

**Background:**

Remote delivery may improve access to pulmonary rehabilitation (PR). Existing studies are largely limited to individuals with COPD, and the interventions have lacked codesign elements to reflect the needs and experiences of people with chronic respiratory disease, their carers/families and healthcare professionals. The aim of this study was, using experience-based codesign (EBCD), to collaborate with people with interstitial lung disease (ILD), their carers/families and healthcare professionals, to codesign a remote PR programme ready for testing in a future study.

**Methods:**

EBCD comprises interviews, stakeholder workshops and codesign meetings. One-to-one videorecorded interviews with purposively selected people with ILD with experience of PR, their carers/families and healthcare professionals, were edited into a 20 min film. The film was shown at three audiorecorded stakeholder feedback events to identify key themes and touchpoints, and short-list key programme components. The programme was finalised at two further codesign workshops.

**Results:**

Ten people with ILD, four carers/families and seven healthcare professionals were interviewed. Participants in the codesign workshops included service-user group: n=14 and healthcare professional group: n=11; joint event: n=21. Final refinements were made with small codesign teams, one comprising three people with ILD and one carer/family member, one with five healthcare professionals. The final codesigned model is a group based, supervised programme delivered by videoconference. Key elements of programme specific to ILD include recommendations to ensure participant safety in the context of desaturation risk, dedicated time for peer support and adaption of the education programme for ILD needs, including signposting to palliative care.

**Conclusion:**

In this EBCD project, a remote PR programme for people with ILD was codesigned by service-users, their carers/families and multidisciplinary healthcare professionals. Future research should explore the feasibility and acceptability of this intervention.

**WHAT IS ALREADY KNOWN ON THIS TOPIC**

- The majority of research investigating remote pulmonary rehabilitation (PR) programmes has involved people with COPD. There are limited data on the nature and efficacy of these programmes in interstitial lung disease (ILD), and such studies include mainly people with idiopathic pulmonary fibrosis, report conflicting results and have methodological limitations. In addition, the interventions used in these studies were not codesigned and may not reflect the needs and experiences of people with ILD, their carers/families or healthcare professionals.

**WHAT THIS STUDY ADDS**

- In this experience-based codesign project, a remote PR programme model for people with ILD was codesigned by service-users, their carers/families and multidisciplinary healthcare professionals. Key elements of the programme specific to people living with ILD include recommendations to ensure participant safety in the context of the risk of exercise-induced oxygen desaturation, dedicated time for peer support and adaption of the education programme for ILD needs, including signposting to palliative care.

**HOW THIS STUDY MIGHT AFFECT RESEARCH, PRACTICE OR POLICY**

- Involving people with ILD, their carers/families and healthcare professionals in the development of a remote PR programme model has generated novel and innovative ideas based on stakeholders’ needs and experience, including ensuring participant safety, dedicated time for peer support and adaption of the education programme for ILD needs, which may optimise translation of research into clinical practice. Future research should investigate the feasibility and acceptability of the intervention.

## Introduction

There is a strong evidence for pulmonary rehabilitation (PR) as a non-pharmacological management strategy for interstitial lung disease (ILD),^1^ and international guidelines recommend that it should be offered to all individuals with ILD.^2–4^ Traditionally, PR comprises a supervised, in-person programme. However, remote programme models, such as telerehabilitation and home-based PR, are increasing in popularity to address problems of limited choice and poor uptake, adherence and completion rates,^5^ in line with the ethos of precision medicine and^6^ and patient-centred care.^7^

The majority of research into remote programmes has involved people with chronic obstructive pulmonary disease (COPD) and there are limited data on the nature and efficacy of these programmes in ILD. For example, of 1904 participants analysed as part of a Cochrane review of telerehabilitation for chronic respiratory disease, only 2 were diagnosed with ILD.^5^ Three randomised controlled trials (RCTs)^8–10^ published since the Cochrane review, and a number of cohort studies^11–15^ that investigated remote models predominantly recruited participants with idiopathic pulmonary fibrosis (IPF) rather than ILD, investigated heterogeneous interventions, reported conflicting results and were limited by small sample sizes. Furthermore, the interventions in these studies were not adapted for ILD. This is relevant as patient advocacy is an essential component of service development and little is known about the needs and preferences of people with ILD regarding remote PR models. Furthermore, national and international guidelines state that PR should be tailored to suit the needs of the service-user,^4 16^ a point emphasised by people living with ILD, who expressed a desire for services designed specifically for their condition^17^ including disease-specific education as well as psychological and palliative support.^18 19^

Qualitative research on the experiences of those who have completed remote PR indicates that people with ILD enjoy videoconference PR^20^ and find supervision by telephone burdensome.^21^ They report that a longer programme as well as the inclusion of social support and ways to engage in exercise after PR would be beneficial.^20 21^ In contrast, healthcare professionals’ opinions and experiences of remote PR models for people living with ILD have not been explored. In addition, no study has codesigned a remote PR programme for ILD, including how it should be adapted for this disease group. Involving people with ILD and healthcare professionals in the development of a remote PR programme may generate innovative ideas and optimise translation of research into clinical practice.^22^ Therefore, this study aimed to, using experience-based codesign (EBCD),^23^ collaborate with people with ILD, their carers/families and healthcare professionals to codesign a remote PR programme ready for testing in a future study.

## Methods

### Design

**Figure 1:**
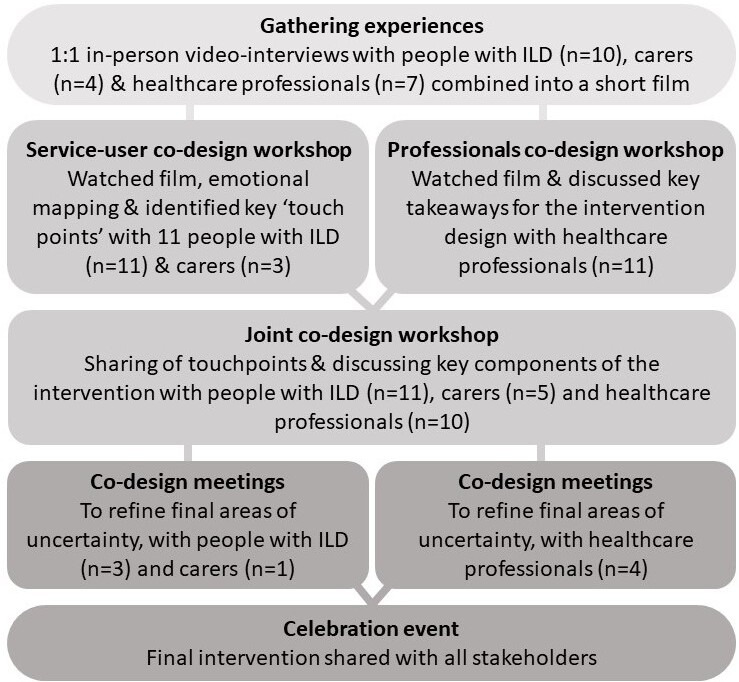
Experience-based codesign process. ILD, interstitial lung disease.

We used EBCD,^23^ an approach that combines design principles, participatory approaches and quality improvement methodology to improve services.^24^ Key stages including gathering experiences, film creation, codesign workshops, small codesign teams and the celebration event are illustrated in figure 1 and described in detail below.

### Participants and sampling

Participants included people affected by ILD, and healthcare professionals with experience relevant to PR. Adults affected by ILD (patients and carers/family; henceforth service-users) were recruited via email by the charity action for pulmonary fibrosis, through in-person invitation within PR services across a health partnership in London, and via our public involvement group for the study. All were required to have experience of PR, regardless of level of completion. Service-users were purposively sampled for diversity relating to sex, ethnicity, respiratory disability (Medical Research Council Dyspnoea Scale 1–3 vs 4–5), and ability to use the internet or not. Additional information, including lung function and prescription of supplemental oxygen, was also recorded.

Healthcare professionals were recruited via email from a health partnership in London and the London PR Network via service leads, and through existing connections with known experts. Professionals required one of the following: at least 1-year experience working in PR, or expertise in PR through national committee activities, research and/or clinical commissioning activities. They were purposefully sampled to represent different professions (physiotherapists vs others) and services (local service vs others).

In all cases, participants were ineligible if they could not provide informed consent, were unable to communicate verbally in English, had a cognitive impairment which would preclude taking part in an interview or group work, or were housebound, which was recommended by our patient representatives due to safety concerns for individuals exercising without in-person supervision.

### Gathering experiences

Interviews were conducted by CMN with service-users and healthcare professionals. The interviews followed topic guides (see online supplemental material) exploring their experiences of PR and preferences relating to remote rehabilitation. Topic guides were created based on existing literature and with input from the patient and public involvement group. Interviews were conducted in-person and videorecorded by a professional filmmaker at Harefield Hospital.

10.1136/bmjresp-2023-002061.supp1 Supplementary data

Interviews were professionally transcribed and analysed by CMN and LJB using conventional content analysis.^25^ Codes and themes were developed primarily inductively from the data. However, there were some deductive influences where construction of themes was influenced by specific areas of uncertainty (eg, delivery format, role of equipment). Themes illustrating suggestions for the adapted model were organised into key ‘touchpoints’: critical moments within the PR journey.

### Film creation

Across two virtual codesign meetings (one with three service-users and one with two healthcare professionals) participants helped the filmmaker and research team to compile a 20 min summary film illustrating the key themes across the touchpoints.

### Codesign workshops

We then held a series of online codesign workshops to codesign the new remote PR intervention. People who took part in the interviews were invited to participate, alongside additional individuals meeting the eligibility criteria if required to supplement the experiences represented. To ensure people affected by ILD with limited internet access and/or computer skills could participate, service-users were offered travel support to join the workshops remotely from the local hospital, with assistance from a member of the PR team. Participants willing but unable to attend the workshops due to illness and/or conflicting responsibilities could also provide input individually.

Workshops were audiorecorded and both facilitators (CMN/LJB) took notes on key discussion points. Content analysis, as described above, was used to summarise key discussion points and decisions made across the codesign workshops.

#### Healthcare professionals’ workshop

Professionals viewed the film and were invited to share their reflections and feedback. The facilitators worked with the group to identify key points relating to the design of the new remote PR intervention design to take forward to the joint workshop.

#### Service-user workshop

Service-users viewed the film and were also invited to respond with their reflections and feedback. This included an emotional mapping exercise with participants to further discuss the key touchpoints, and the implications of the themes from the interviews for the design of the new remote PR intervention. Suggestions were combined with the results of the staff event to inform the final joint workshop.

#### Joint workshop

Both professionals and service-users were invited to participate together in the final codesign workshop. The facilitators presented areas of agreement and discrepancy from the previous sessions. Participants were invited to provide further feedback, and discussion particularly focused on resolving areas of disagreement and uncertainty to help further refine the proposed intervention.

### Small codesign teams

Based on the outcomes of the codesign workshops, the research team worked closely with smaller codesign teams to finalise the details of the new remote model of PR for people with ILD. This included an additional workshop each with a smaller group of service users and professionals, to ensure the final model was true to the findings of the codesign meetings and to resolve final uncertainties.

### Celebration event

The final model was shared with all participants in a virtual celebration event. Participants were informed of the next stages for the work and invited to continue their involvement in subsequent developments.

### Patient and public involvement

People affected by ILD were involved throughout as members of the study team. Examples of contributions included protocol design, codesigning the interview topic guides (presented in online supplemental material), participating in the small codesign teams, supporting analysis and interpretation, and contributing to this publication. Additional information on patient and public involvement is reported in the appendix using the GRIPP2-short-form checklist.^26^

### Reflexivity

CMN is a mixed-methods researcher with a background in PR. CMN had an existing relationship with one ILD participant who had taken part in a previous study and five healthcare professional participants whom she had previously worked with. LJB is an applied health researcher with a background in psychology and palliative care, and an experienced qualitative researcher. LJB had no existing relationship with any of the participants with ILD. She knew three of the PR practitioners and the palliative care specialist through previous research collaborations. To reduce the impact of these existing relationships, prior to conducting the interviews (conducted by CMN), CMN and LJB actively discussed how these relationships may influence the interview conduct. During the interviews, which were observed by a film maker who was independent to the research team, CMN kept a reflexive diary to reflect on and understand her role during the interview. After the interviews, CMN discussed the interviews with LJB. During the analysis phase, CMN and LJB actively discussed how their experiences might influence the analysis and ensured they frequently returned to the data and sought the input of public members to strengthen interpretive rigour.

## Results

The interviews were conducted in November and December 2021, and the two virtual codesign meetings to develop the film were held in January 2022. The codesign workshops were all held in May 2022. Two additional codesign meetings to finalise the intervention were held in June 2022. Interview participants are described in table 1, and an overview of participation across each stage of the codesign process is shown in box 1. All interviewees took part in at least one component of the codesign process. An additional three service-users and five healthcare professionals who were not interviewed took part in the codesign process.

**Table 1:** Characteristics of interviewees

Service-users (n=10)*	Baseline characteristics
Female sex	6 (60%)
Age	72 (8)
Ethnicity	
White British	5 (50%)
Indian	3 (30%)
Black Caribbean	1 (10%)
Other White background	1 (10%)
ILD disease category	
IPF	5 (50%)
CT-ILD	2 (20%)
Sarcoidosis	2 (20%)
Antisynthetase syndrome associated ILD	1 (10%)
Forced vital capacity (L)†	2.18 (0.85)
Forced vital capacity (% predicted)†	68.0 (25.5)
Transfer of the lung for carbon monoxide (mmol/min/kPa)‡	3.58 (1.35)
Transfer of the lung for carbon monoxide (%predicted)‡	55.5 (23.5)
Prescribed long-term oxygen therapy	2 (20%)
Prescribed ambulatory oxygen therapy	5 (50%)
MRC	
≤3	4 (40%)
≥4	6 (60%)
Able to use the internet	6 (60%)
Previously completed PR	9 (90%)
PR programme model experience	
In-person only	6 (60%)
Video-conference PR only	2 (20%)
Telephone support PR only	1 (10%)
Videoconference and telephone support PR	1 (10%)
**Healthcare professionals (n=7)**	
Female sex	4 (60%)
Profession	
Physiotherapist	6 (86%)
Physiotherapy assistant	1 (14%)
PR service	
Harefield PR unit	4 (60%)
London PR network	3 (40%)

Data are reported as number (percentage) or mean (SD).

*Four spouses were also interviewed alongside their partner but demographic data were not collected.

†Forced vital capacity data were only available for eight participants.

‡Transfer factor of the lung for carbon monoxide data were only available for five participants.

ILD, interstitial lung disease; IPF, idiopathic pulmonary fibrosis; MRC, Medical Research Council Dyspnoea Scale; PR, pulmonary rehabilitation.

Box 1 **Attendees at the feedback events and codesign workshops**

**Codesign workshops to develop the film**

- Person with ILD n=3.
- Carer/family of person with ILD n=1.
- Physiotherapist n=2.

**Feedback events**

Service-user event

- Person with ILD n=11.
- Carer/family of person with ILD n=3.

Healthcare professional event

- Physiotherapist n=7.
- Physiotherapy assistant n=2.
- Nurse n=1.
- Occupational therapist n=1.

Joint event

- Person with ILD n=11.
- Carer/family of person with ILD n=5.
- Physiotherapist n=6.
- Physiotherapy assistant n=2.
- Nurse n=1.
- Occupational therapist n=1.

**Codesign workshops to finalise the intervention**

Service-user workshop

- Person with ILD n=3.
- Carer/family of person with ILD n=1.

Healthcare professional workshop

- Physiotherapist n=1.
- Physiotherapy assistant n=1.
- Nurse n=1.
- Occupational therapist n=1.
- Palliative care consultant n=1*.

*The palliative care consultant was unable to attend the workshop and her opinions were sought at a separate meeting.ILD, interstitial lung disease.

Service-users described the impact of living with ILD and how PR can help them manage their condition. After completing an in-person PR programme, one participant described: ‘*I did feel I had a pep in my step, I could go up the stairs without being so breathless, I was still breathless, but I could feel the difference’* (Service-user (SU) 07). Participants agreed that a remote PR programme could be beneficial; as one participant summarised: ‘*It would be a good idea, would be a really good idea. Because (a) it cuts down on travel. Plus, it cuts down on stress, you're in the comfort of your home.’* (SU10)

**Figure 2:**
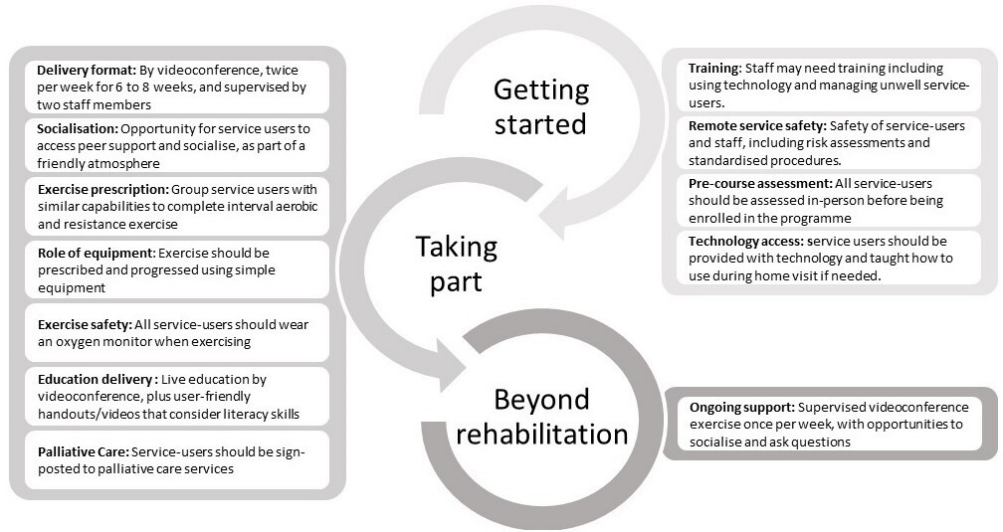
Key touchpoints and suggestions for the remote rehabilitation.

**Figure 3:**
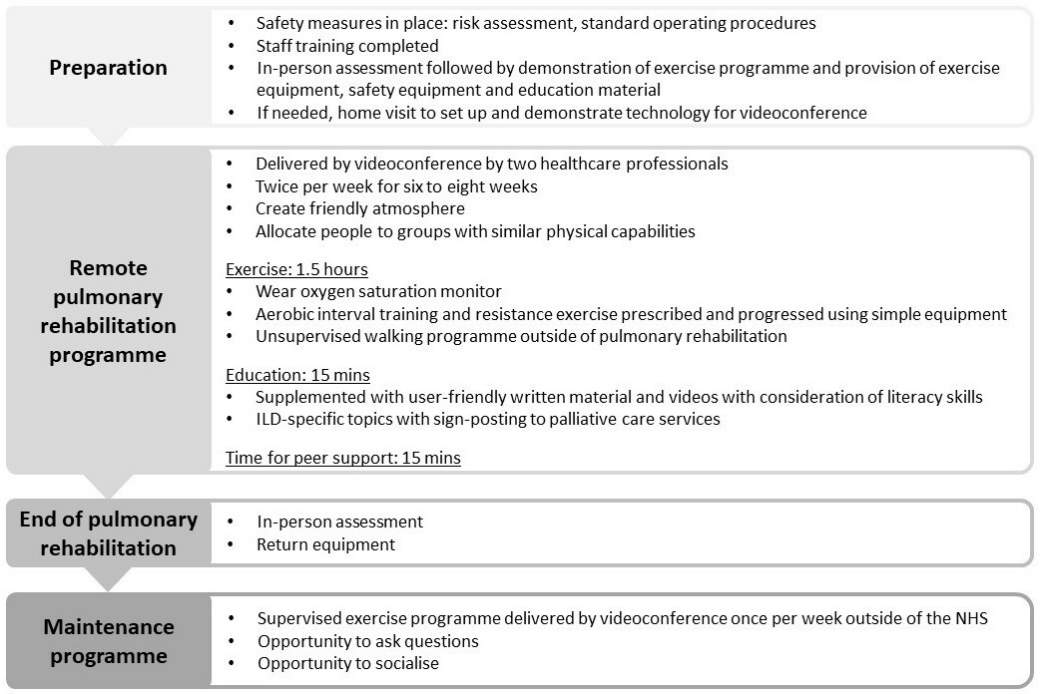
Final intervention design. ILD, interstitial lung disease; NHS, National Health Science.

Key touchpoints in the rehabilitation journey included: ‘getting started’ as people prepared for participation in rehabilitation, ‘taking part’ in rehabilitation and ‘beyond rehabilitation’, where people prepared for continued self-management beyond the end of the structured programme. Elaboration on these touchpoints and their preferences for a remote programme are described below and in figure 2. Illustrative quotes from participants are shown in table 2, and the final intervention is outlined in figure 3.

**Table 2:** Touchpoints, themes and illustrative quotes

**Getting started**		
Remote service safety	Q1	‘I wouldn't feel unsafe about any of it. I feel sure that my wife is always there anyway, she would keep an eye on me as she does. And if she thinks that I'm overdoing it, or thinks I'm getting a bit too tired or exhausted, and she stops me doing whatever I'm doing.’ (SU09)
	Q2	‘You give them an alarm, you know, like the fall’s pendant, you give them that. And then if they are in distress, they can press that.’ (Healthcare professional (HCP) 05)
Training	Q3	‘But what we don't know is the best way to prescribe [exercise] and there’s no gold standard [way to do this for remote PR], as far as I know’ … ‘What we end up doing is’ … ‘a bit of trial and error, where we kind of give it to our patients and then say, how does this work for you?.’(HCP03)
	Q4	‘Creating a resource book for physios as well, so that they know how to troubleshoot issues which may come through during delivering a PR class. So, for example, how to set up meetings, how to re-invite somebody if they've lost the link‘ … ‘The second thing would be how to manage crisis situations’ … ‘if a patient collapsed on the screen, how would you manage that?’ (HCP05)
Precourse assessment	Q5	‘And making sure we have that face-to-face interaction initially, where we can screen any risk factors like say a balance problem, or is there a cardiac issue?’ (HCP07)
	Q6	‘It’s important because you need the assessment to know your level of fitness before you start the programme.’(SU07)
Technology access	Q7	‘An ideal rehab programme would be that a patient comes [in] for an assessment, we do a thorough assessment and then give out the equipment, whatever they need for the programme. If you think the patient is not tech savvy or the patient has more needs, then one of the team members can go out to the patient’s home, check the area, teach them how to use the computer, what buttons to use, how to use the sats [oxygen] probe et cetera.’ (HCP05)
**Taking part**		
Delivery format	Q8	‘But one thing that it would need would be a physiotherapist on the other end to motivate you. And to see you’re doing okay. So probably in Zoom with a group of people and one physio or two.’ (SU10)
	Q9	‘Videoconference is my preference because it’s the closest you can have in mimicking a face-to-face group.’ (HCP05)
Socialisation	Q10	‘I think as well as the exercise aspect of it, it would probably be good to have a social support type of group’ … ‘It’s nice to have someone to talk to and then you can just say, ohh well, I’m not having a very good week’ … ‘And sometimes, you know, you can help to motivate each other and also provide support.’ (SU02)
Exercise prescriptions	Q11	‘So, if you have a class, which is pitched at the same level, they tend to perform better compared to a mixed class where there is always somebody lagging back, because they will feel frustrated that they're not able to keep up and the higher-level people will be frustrated, because it takes longer for the slower person to catch up.’ (HCP05)
Role of equipment	Q12	‘I’d prefer for money to be spent on NHS staff advising us rather than buying equipment’ … ‘As people have mentioned, there are issues like whether people haven't got the space or whether after the equipment is withdrawn will they continue doing the exercise.’ (SU03)
	Q13	‘I feel that all you need are the bands [therabands] and the use of a chair, just an ordinary chair. That is quite sufficient.’ (SU13)
	Q14	‘I came across a conversion chart using pints of milk’ … ‘I think I think 6 pints is about 3.6 kilograms from memory’ … ‘so let’s not undervalue what we have already around the house.’ (HCP04)
Exercise safety	Q15	‘I have a oxygen monitor’…. ‘my [oxygen] concentration levels go down very rapidly. Frighteningly low.’ (SU04)
Education delivery	Q16	‘I think Zoom would be the best one, because with recorded videos, you can watch them. But if you have questions, you usually want to ask or get answer straight away.’ (SU02)
	Q17	‘I would like leaflets as well. Because when the physio taught me how to breathe, for my cough, you take it in at that time, but after a little while, you forget’. (SU10)
Palliative care	Q18	‘I think one of the big topics is end of life care, it’s a very sensitive topic. And, again, also needs to be delivered in a very sensitive way.’ (HCP04)
**Beyond rehabilitation**		
Ongoing support	Q19	‘I'd like them not to stop it’ … ‘I think you need the encouragement to carry on. I didn't hear from them [after the programme]. So, I felt a bit lost.’ (SU06)
	Q20	‘It would be great if we can carry on with the maintenance program and online at least once a week. And where, you know, people can meet not only for the exercise, but also the socialisation part’ … ‘you know, they have some level of support from a healthcare professional.’ (HCP06)

### Getting started

Themes from the interviews discussed here focused on remote service safety, training, precourse assessment and technology access. The majority of service-users would feel safe taking part in a remote programme. All participants stressed the importance of ensuring it was safe by implementing measures including risk assessments, standard operating procedures and emergency equipment for example, personal alarm (quote (Q)1; Q2). Healthcare professionals highlighted that training may be required to deliver the programme effectively and safely, including skills for prescribing and progressing exercise (Q3), using technology and managing an unwell service-user (Q4).

Participants emphasised the importance of an in-person assessment prior to starting the programme for safety screening and to assess service-users’ ability, set goals, demonstrate the exercise programme and provide exercise equipment and education material (Q5, Q6). For service-users unable to use the Internet, participants stated that a staff member should undertake a home visit to supply and/or teach the individual how to use the videoconference equipment and do the exercise programme (Q7).

### Taking part

Themes relating to taking part included programme delivery format, opportunities for socialisation, exercise prescriptions, the role of equipment, exercise safety, education delivery and coverage of palliative and end-of-life care.

Participants agreed that the programme should be group-based and supervised by videoconference (Q8, Q9) by two staff members, in line with PR guidelines, that is, twice per week for 6–8 weeks^2–4^ in order to ensure alignment with evidence-based practice and appropriate reimbursement. Service-users also highlighted the importance of creating a social atmosphere with time to engage in peer support, especially as the programme would be delivered by videoconference, using initiatives including group introductions, ice breakers and dedicated time to socialise (Q10).

Participants recognised the importance of the exercise component of PR and the inclusion of both aerobic and resistance training. Healthcare professionals reported that prescribing and progressing exercise by videoconference is challenging. Therefore, it was proposed that service-users should be allocated to a group with similar levels of breathlessness or physical capabilities (Q11). Service-users strongly opposed the idea of specialist exercise equipment, for example, cycle ergometers, treadmills, because it would take up space in their homes and may pose unnecessary financial burden on the service (Q12). Therefore, it was proposed that aerobic exercise could be prescribed using the principles of high-intensity interval training as it would be difficult to prescribe continuous aerobic exercise at home without specialist equipment. Accordingly, the duration of the exercise component of the programme would need to be extended to account for this, for example, 90 vs 60 min. All stakeholders agreed that this should be supplemented by an unsupervised walking programme undertaken independently of PR but recorded on an app (eg, Strava), in order to improve endurance capacity. All participants agreed that simple exercise equipment, for example, free weights that could be supplied at the pre-PR assessment, or readily available equipment at home (eg, chairs, water bottles) were acceptable (Q13). This would enable the pragmatic prescription of resistance exercise in line with international guidelines^27^ (Q14).

There were mixed opinions on wearing a peripheral oxygen saturation monitor because of the risk of service-users focusing on oxygen saturation levels rather than how they feel and overmedicalising exercise. However, the majority of participants stated that the monitors should be worn by service-users for safety, that is, the risk of profound exercise-induced oxygen desaturation and rapid disease trajectory for select individuals (Q15).

Participants recognised the importance of the education component of PR and indicated that it should be delivered ‘live’ by videoconference but supplemented by user-friendly written material and videos tailored for variation in literacy skills (Q16, Q17). Service-users indicated that the education programme should be specific to ILD. Participants highlighted topics such as symptom management, oxygen therapy and mood management, as well as disease progression and palliative care. Healthcare professionals recognised the latter as an important subject but did not think they had the specialist skills to effectively teach it. The palliative care consultant did not believe PR provided the most appropriate environment to discuss this sensitive issue, but suggested service-users should be sign-posted to palliative care services as required (Q18).

#### Beyond rehabilitation

For beyond rehabilitation, the main theme discussed focused on ongoing support. Service-users stated a preference for an ongoing programme and access to a healthcare professional to seek support but acknowledged it may not be possible due to health service constraints (Q19). Healthcare professionals recognised the importance of offering a maintenance programme to enable service-users to continue to access supervised exercise training (Q20), and suggested this could be offered by a third party, for example, a community exercise programme.

## Discussion

We codesigned a remote PR programme for ILD with service users and multidisciplinary healthcare professionals. Key elements of the remote programme specific to ILD include recommendations to ensure participant safety in the context of the risk of exercise-induced oxygen desaturation, dedicated time for peer support and adaption of the education programme for ILD, with specific recommendations for palliative care.

Given the risk of exercise-induced oxygen desaturation and the rapid disease trajectory for select individuals with ILD, participants highlighted the importance of proactive measures to ensure participant safety. For example, it was recommended that peripheral oxygen monitors should be worn when exercising and oxygen saturations recorded regularly throughout the exercise session, in addition to the development of protocols to manage unwell patients and emergency situations. Only two small RCTs (n=21 fibrotic ILD,^9^ n=29 IPF^10^) which involved remote monitoring systems recorded peripheral oxygen saturation during exercise but did not report the results nor adverse event data, which limits interpretation of the safety of remote programmes in ILD.

Similar to previous qualitative research,^20^ the importance of the social aspect of the programme was emphasised by people with ILD, in particular having an opportunity to access peer support. It is noteworthy that previous studies of remote programmes in ILD did not include this in the intervention,^8–15^ therefore, its impact has not been investigated. For people with ILD, peer support provides a way to connect with other people with the same disease and is an enabler of exercise.^20^ While valuing peer support is common across respiratory conditions, this aspect might be particularly important for people with ILD given that the condition is less common and opportunities to meet people with similar experiences may be infrequent. Dedicated time for this was, therefore, included in the final programme.

As reported in previous research on traditional in-person PR programmes,^18 19^ participants highlighted the importance of adapting the education programme for ILD, with consideration of variation in literacy, including, for example, disease pathophysiology and progression, medical management and oxygen therapy. The importance of education about palliative care was recognised by all participants, and aligns with calls to integrate this approach into routine ILD care. While palliative care needs are common across respiratory illnesses, the shorter average life expectancy for select individuals postdiagnosis (compared with, eg, COPD) may make introductions to palliative care particularly relevant. However, it was agreed that PR may not be the most appropriate setting for this sensitive topic and that interested individuals could be sign-posted to specialist services for further information and/or care. While previous studies on remote PR programmes in ILD included education,^9 10 13–15^ only 1 trial involving 29 participants^10^ provided IPF-specific content. This trial did not address palliative care needs nor evaluate this aspect of the programme.

Other important elements of the programme highlighted by participants were the programme structure and recommendations for the exercise component. Regarding structure, participants emphasised the importance of a supervised, ‘live’ programme underpinned by evidence-based practice. Therefore, the final programme involved an in-person assessment and videoconference PR delivered in line with PR guidelines.^2 16 28^ This is contrast to previous research of remote PR programmes in ILD where the exercise component was predominantly unsupervised^8 9 11–15^ or supervised using a virtual physiotherapist^10^ and delivered on a telerehabilitation platform,^9–12^ Wii Fit^8^ or at home.^10 13–15^

Regarding the exercise component, participants recommended allocating service-users with similar levels of functional ability to the same group, the use of simple exercise equipment available in the home (eg, chair) supplemented with free weights or elastic bands provided by the PR service, the prescription of high-intensity interval aerobic training, and an unsupervised session involving continuous aerobic exercise. These exercise recommendations have not been explored in previous studies of remote PR programmes in ILD, therefore, their feasibility, acceptability and efficacy should be investigated.

### Strengths and limitations

Our sample included people with diverse ethnicities, levels of socioeconomic deprivation, ILD diagnoses, disease stage and supplemental oxygen prescription, as well as diverse professional roles, increasing the transferability of our findings. Similarly, we supported participants who were unable to use the Internet to attend the online meetings, which is important as 31% of PR service-users have never accessed the Internet.^29^ We also included participants with experiences of a variety of models of PR, and accommodated the flexible involvement of participants at each stage of the EBCD process due to difficulties will illness and/or other commitments. Including service-user and professional stakeholders in the codesign process ensured that a range of preferences and concerns were explored that may not have been comprehensively captured by one group. For example, it was service-users that particularly championed the importance of socialisation, while the difficulties with exercise prescription were only raised by professionals.

Despite these strengths, experiences may particularly reflect those of people based in London and the South-East of the UK, and more work is required to understand the needs of people with ILD who are unable to communicate in English and who are housebound. In addition, we excluded people who did not have any experience of PR, which may bias our results. The design of the programme was also constrained in some ways by the accepted PR definition,^2 16 28^ as participants agreed this would be important for reimbursement of services. However, the EBCD approach enabled participants to prioritise the content and delivery most suited to people with ILD, and emphasise the importance of the ‘beyond rehabilitation’ stage of the intervention. Using a codesign approach may also result in an intervention that is more likely to be feasible and acceptable, supporting translation from the research to clinical setting.

## Conclusion

A remote PR programme model for people with ILD was codesigned with service-users, their carers/families and multidisciplinary healthcare professionals. Key elements included specific recommendations for the exercise component as well as ensuring participant safety and dedicated time for peer support. Future research should explore the feasibility and acceptability of this intervention.

## Data Availability

Data are available on reasonable request.
