# Supplementary material for: Remote pulmonary rehabilitation for interstitial lung disease: developing the model using experience-based codesign
Source: BMJ Open Respir Res. 2024 Feb 20;11(1):e002061. doi: 10.1136/bmjresp-2023-002061 (PMC10882322; doi:10.1136/bmjresp-2023-002061)
Supplement: Supplementary data [file bmjresp-2023-002061supp001.pdf]

Remote pulmonary rehabilitation for interstitial lung disease: Developing the model using experience-based co-design

Supplementary material

METHODS: Topic guide for people living with ILD their carers/family

Pulmonary rehabilitation experience	
**1. Can you tell me about your experience of pulmonary rehabilitation?**	
Home-based pulmonary rehabilitation – general views	
**2. Can you tell me what you think about doing a rehabilitation programme at home?**	
Home-based pulmonary rehabilitation – content	
**3. What do you think a home-based rehabilitation programme should include?**	How important or not do you think exercise as part of the home programme would be?<br>What type of exercise might you feel confident doing as part of a home-based rehabilitation programme?<br>How important or not do you think using exercise equipment would be?<br>If you had to do a rehabilitation programme at home, what might help you to feel safe when exercising? (Prompt: emergency call bell, assessment, oxygen monitor, ability to contact staff)<br><br>How important or not do you think education as part of the home programme would be?<br>What would you be interested in learning about?<br>What are the topics that are most important to you? (Prompt: managing breathing, pacing, nutrition, exercise e.g. walking)<br>How might you like an education programme to be delivered? (Prompt: booklet, recorded videos, live session via video-conference)
Home-based pulmonary rehabilitation – delivery and logistics	
**4. How do you think a home-based programme should be delivered?**	If you had to do a rehabilitation programme at home, how would you like to do it? (*Prompts: 'Zoom' or video-conference classes, support by telephone, home visits)*

Brighton LJ, *et al. BMJ Open Respir Res* 2024; 11:e002061. doi: 10.1136/bmjresp-2023-002061

**5. How often do you think the sessions for a home-based programme should be?**	Prompt: Traditional pulmonary rehabilitation runs twice a week
**6. How long do you think a home-based programme should run for?**	Prompt: Traditional pulmonary rehabilitation lasts six to eight weeks
**7. What do you think about having an assessment with a pulmonary rehabilitation professional before you start your home-based programme?**	
**8. What are your views on whether home-based programmes should be supervised by a pulmonary rehabilitation professional?**	Prompts: telephone, video-conference, in-person
Barriers and facilitators	
**9. What do you think would encourage people with ILD to take part in a home-based rehabilitation programme?**<br><br>**10. What do you think would stop people with ILD from taking part in a home-based rehabilitation programme?**<br><br>**11. What do you think would encourage people with ILD to keep attending the whole programme, and what would stop them attending the whole programme?**<br><br>**12. What would encourage you to keep exercising when the home-based rehabilitation programme ended?**	
End of interview	
**13. Is there anything else you would like to tell me before we finish?**	Thank you

METHODS: Topic guide for healthcare professional participants

Pulmonary rehabilitation experience	
**1. Can you tell me about your experience delivering pulmonary rehabilitation?**	
Home-based pulmonary rehabilitation – experience	
**2. Can you tell me about your experience delivering home-based pulmonary rehabilitation?**	Prompts: virtual, non-virtual
Home-based pulmonary rehabilitation – content	
**3. What do you think a home-based rehabilitation programme should include?**	How important or not do you think exercise as part of the home programme would be?<br>What type of exercise might you feel confident prescribing as part of a home-based rehabilitation programme?<br>How would you find prescribing and progressing a home-based exercise programme?<br>How important or not do you think using exercise equipment would be?<br>If you had to deliver a rehabilitation programme at home, what might help you to feel that your patients would be safe when exercising? (Prompt: emergency call bell, assessment, oxygen monitor, ability to contact other staff, ability to contact patient)<br><br>How important or not do you think education as part of the home programme would be?<br>What topics do you think people with ILD would be be interested in learning about? (Prompt: managing breathing, pacing, nutrition, exercise e.g. walking)<br>How might you like to deliver an education programme? (Prompt: booklet, recorded videos, live session via video-conference)<br>What teaching styles do you think might work best for home-based programmes?
**4. What are your views on whether or not home-based programmes need to be tailored to suit the needs of people with ILD?**	What would this look like?
Home-based pulmonary rehabilitation – delivery and logistics	
**5. How do you think a home-based programme should be delivered?**	If you had to deliver a rehabilitation programme at home, how would you like to do it? (Prompts: 'Zoom' or video-conference classes, support by telephone, home visits)

**6. How often do you think the sessions for a home-based programme should be?**	Prompt: Traditional pulmonary rehabilitation runs twice a week
**7. How long do you think a home-based programme should run for?**	Prompt: Traditional pulmonary rehabilitation lasts six to eight weeks
**8. What do you think about patients having an assessment with a pulmonary rehabilitation professional before they start their home-based programme?**	
**9. What are your views on whether home-based programmes should be supervised by a pulmonary rehabilitation professional?**	Prompts: telephone, video-conference, in-person<br>How often?
Training	
**10. What training do you think would help pulmonary rehabilitation professionals feel confident to deliver a high-quality home-based rehabilitation programme?**	
End of interview	
**11. Is there anything else you would like to tell me before we finish?**	Thank you

Brighton LJ, *et al. BMJ Open Respir Res* 2024; 11:e002061. doi: 10.1136/bmjresp-2023-002061

METHODS: Patient and public involvement reported using the GRIPP2-short-form checklist [1]

Section and topic	Item
**1: Aim**<br>*Report the aim of PPI in the study*	The aim of PPI throughout this project was to: ensure acceptable and appropriate research processes where patient/carers would be participating, include patient/carer voices throughout the co-design process and improve clarity of dissemination.
**2: Methods**<br>*Provide a clear description of the methods used for PPI in the study*	We used remote methods (via Teams, email and telephone) to conduct our PPI activities, due to restrictions imposed by the Covid-19 pandemic. Involvement was flexible, with some members being more involved in some study components than others.
**3: Study results**<br>*Outcomes—Report the results of PPI in the study, including both positive and negative outcomes*	CN was the PPI lead which involved recruitment of the team and replacement of members no longer able to participate. Following the involvement of one PPI members in development of the original project application, an additional two PPI members joined the team. During the study, one PPI member left the group due to illness and was replaced by another individual and his carer. Sadly, between study completion and dissemination, one of the PPI members died.<br><br>The PPI members reviewed the study protocol, plain English project summary, attended PPI meetings and were invited to project meetings where PPI input was required. Contributions to specific project components to date are as follows:<br><br>Protocol<br>-Exclusion of people who were housebound<br>-Inclusion of people unable to use the Internet<br><br>*Interviews and film creation*<br>- Assisting with the development of the qualitative interview topic guides<br>- Assisting with the analysis of the qualitative interviews<br>- Assisting with the development of the script for the film<br>- Reviewing and commenting on the film content<br><br>*Small co-design team workshops*<br>- Reviewing and reflecting on the large co-design workshops<br>- Revising and refining the final intervention<br><br>*Dissemination*<br>- Co-authoring the scientific abstract<br>- Co-authoring this manuscript<br>- After this manuscript is published, we will work with our PPI team to disseminate the results through plain English summaries.<br><br>*Future research*

Section and topic	Item
	- One of the PPI members has joined a new PPI team and supported the submission of a grant application to investigate the feasibility and acceptability of the intervention developed as part of this project
**4: Discussion and conclusions**<br>*Outcomes—Comment on the extent to which PPI influenced the study overall. Describe positive and negative effects*	There have been multiple positive outcomes from PPI in our study, in line with our aims:<br>*Ensure acceptable and appropriate research processes where patient/carers would be participating:*<br>Involvement of our PPI members in reviewing the study protocol improved the acceptability of the study to participants e.g. inclusion of people who don't use the Internet.<br>Their assistance with the plain English summaries ensured that the information was clear and readable by participants from all backgrounds.<br>Their help with developing the topic guide ensured participants understood the questions (e.g. rephrasing of questions to make them simpler), and that the questions were relevant.<br>*Include patient/carer voices throughout the co-design process:*<br>PPI members ensured that the patient and carer voice was accurately represented in thematic analysis and film and not limited to researcher interpretations. This included reinforcement of safety when exercising (and ideas for increasing safety), the role of the carer, as well as the importance of inclusion and how to support people who live alone.<br>The work of the PPI team in the small co-design meetings ensured that the final intervention reflected the needs of the people living with ILD and their carers/ family.<br>Having PPI members attend the PPI and project meetings ensured opportunities to relate emerging findings to their real-life experiences (e.g. challenges when exercising remotely without equipment and how they overcame them).<br>*Improve clarity of dissemination:* By commenting on and co-authoring the scientific abstract and manuscript, PPI members have increased clarity. They have also agreed to help write plain English summaries.
**5: Reflections/critical perspective**<br>*Comment critically on the study, reflecting on the things that went well and those that did not, so others can learn from this experience*	We feel that this project benefited greatly from PPI (as outlined above). It was beneficial to have multiple PPI members engaged at the start of the project, as this gave flexibility for more or less involvement from individuals at different stages, in line with their interests, other commitments, and/or health.<br>Our PPI members have commented that they felt valued as part of the project team, leading to more rewarding and satisfying involvement.

Brighton LJ, *et al. BMJ Open Respir Res* 2024; 11:e002061. doi: 10.1136/bmjresp-2023-002061

References

1 Staniszewska S, Brett J, Simera I, et al. GRIPP2 reporting checklists: tools to improve reporting of patient and public involvement in research, *BMJ* 2017;358.
